# Antimicrobial and Chemotactic Activity of Scorpion-Derived Peptide, ToAP2, against *Mycobacterium massiliensis*

**DOI:** 10.3390/toxins10060219

**Published:** 2018-05-30

**Authors:** Lázaro M. Marques-Neto, Monalisa M. Trentini, Rogério C. das Neves, Danilo P. Resende, Victor O. Procopio, Adeliane C. da Costa, André Kipnis, Márcia R. Mortari, Elisabeth F. Schwartz, Ana Paula Junqueira-Kipnis

**Affiliations:** 1 Laboratório de Imunopatologia das Doenças Infecciosas, Instituto de Patologia Tropical e Saúde, Pública, Universidade Federal de Goiás, Goiânia CEP 74605-050, Goiás, Brazil; lazaromoreiraneto@hotmail.com (L.M.M.-N.); monalisatrentini@gmail.com (M.M.T.); rogeriocdasneves@hotmail.com (R.C.d.N.); danilo.resende2@hotmail.com (D.P.R.); victor.goler16@gmail.com (V.O.P.); adeliane.castrodacosta@gmail.com (A.C.d.C.); 2 Laboratório de Bacteriologia Molecular, Instituto de Patologia Tropical e Saúde Pública, Universidade Federal de Goiás, Goiânia CEP 74605-050, Goiás, Brazil; andre.kipnis@gmail.com; 3 Laboratório de Neurofarmacologia, Instituto de Ciências Biológicas, Universidade de Brasília, Brasília 70919-970, DF, Brazil; mamortari@gmail.com (M.R.M.); beth.ferroni@gmail.com (E.F.S.)

**Keywords:** antimicrobial peptide, mycobacteria inhibition, *Mycobacterium*, monocyte, neutrophil

## Abstract

*Mycobacterium massiliense* is a rapid growing, multidrug-resistant, non-tuberculous mycobacteria that is responsible for a wide spectrum of skin and soft tissue infections, as well as other organs, such as the lungs. Antimicrobial peptides had been described as broad-spectrum antimicrobial, chemotactic, and immunomodulator molecules. In this study we evaluated an antimicrobial peptide derived from scorpion *Tityus obscurus* as an anti-mycobacterial agent in vitro and in vivo. Bioinformatics analyses demonstrated that the peptide ToAP2 have a conserved region similar to several membrane proteins, as well as mouse cathelicidin. ToAP2 inhibited the growth of four *M. massiliense* strains (GO01, GO06, GO08, and CRM0020) at a minimal bactericidal concentration (MBC) of 200 µM. MBC concentration used to treat infected macrophages was able to inhibit 50% of the bacterial growth of all strains. ToAP2 treatment of infected mice with bacilli reduced the bacterial load in the liver, lung, and spleen, similarly to clarithromycin levels (90%). ToAP2 alone recruited monocytes (F4/80^low^ Gr1), neutrophils (F4/80^−^ Gr1), and eosinophils (F4/80+ Gr1+). ToAP2, together with *M. massiliense* infection, was able to increase F4/80^low^ and reduce the percentage of F4/80^high^ macrophages when compared with infected and untreated mice. ToAP2 has in vitro anti-microbial activity that is improved in vivo due to chemotactic activity.

## 1. Introduction

One of the main goals of modern medicine is the search for new antimicrobials, since outbreaks of extremely resistant bacteria are becoming more common. *Mycobacterium massiliense* (Mycmas) is a fast growing mycobacteria belonging to the *Mycobacterium abscessus* complex [1], which was earlier described as causing infections related to outbreaks mainly associated with skin and soft tissue infections [2,3]. However, this infection profile changed, as described by Sfeir et al. (2018), and nowadays infections occurring in the respiratory tract have become the most worrisome cases [4].

The bacteria of this complex have resistance profiles to glutaraldehyde, widely used for disinfection in hospitals, as well as resistance to the main antimicrobial agents used to treat tuberculosis [3]. Due to this, the treatment consists of a combination of clarithromycin (CLR), amikacin (AMK), cefoxitin (FOX), and/or imipenem (IPM), which may present toxic effects to the liver, kidneys, blood, and skin, and occur mainly when administered for long periods [5].

In in vitro tests, several antimicrobial peptides (AMPs) have been described as having a broad antimicrobial spectrum, acting not only against Gram-positive and -negative bacteria, but also against fungi and virus [6,7,8]. In mammals, AMPs are more effective against the microorganisms at the site where they are secreted, for example, β-defensins are present in the skin and are particularly effective against *Staphylococcus aureus* [9]. AMPs are present in several species of different kingdoms, for example, in snake [10], wasp [11], bee [12], and scorpion venoms [13], and have also demonstrated action against several microorganisms, including human pathogens.

In addition to their antimicrobial activity, AMPs have been described as having a broad ability to modulate the immune response [14]. They may attract monocytes, macrophages, neutrophils, and epithelial cells, as well as modulate or stimulate cytokine production [15,16]. For example, human or mouse cathelicidins, as well as peptides derived from arthropods, have been described to recruit monocytes, macrophages, and T cells, in addition to have immunoregulatory activity, inhibit LPS response, and promote wound healing [17,18]. Accordingly, the antimicrobial activity is enhanced by the ability to interact with host cells by promoting or improving the immune response against infection.

Recently it has been shown that the ToAP2 peptide, derived from the venom of the scorpion *Tityus obscurus* and belonging to the group of peptides without disulfide bridge (NDBP), subfamily 3, provided activity against *Cryptococcus neoformans* and *Candida* spp., as well as avoid *Candida albicans* biofilm formation [19]. Thus, the objective of this study was to determine the antimicrobial activity, both in vitro and in vivo, of the ToAP2 peptide against *Mycobacterium massiliense* and to evaluate the chemotactic activity of these peptides, which is directly related to the therapeutic potential.

## 2. Results

### 2.1. ToAP2 Secondary Structure and Similarity with Transmembrane Proteins

**Figure 1:**
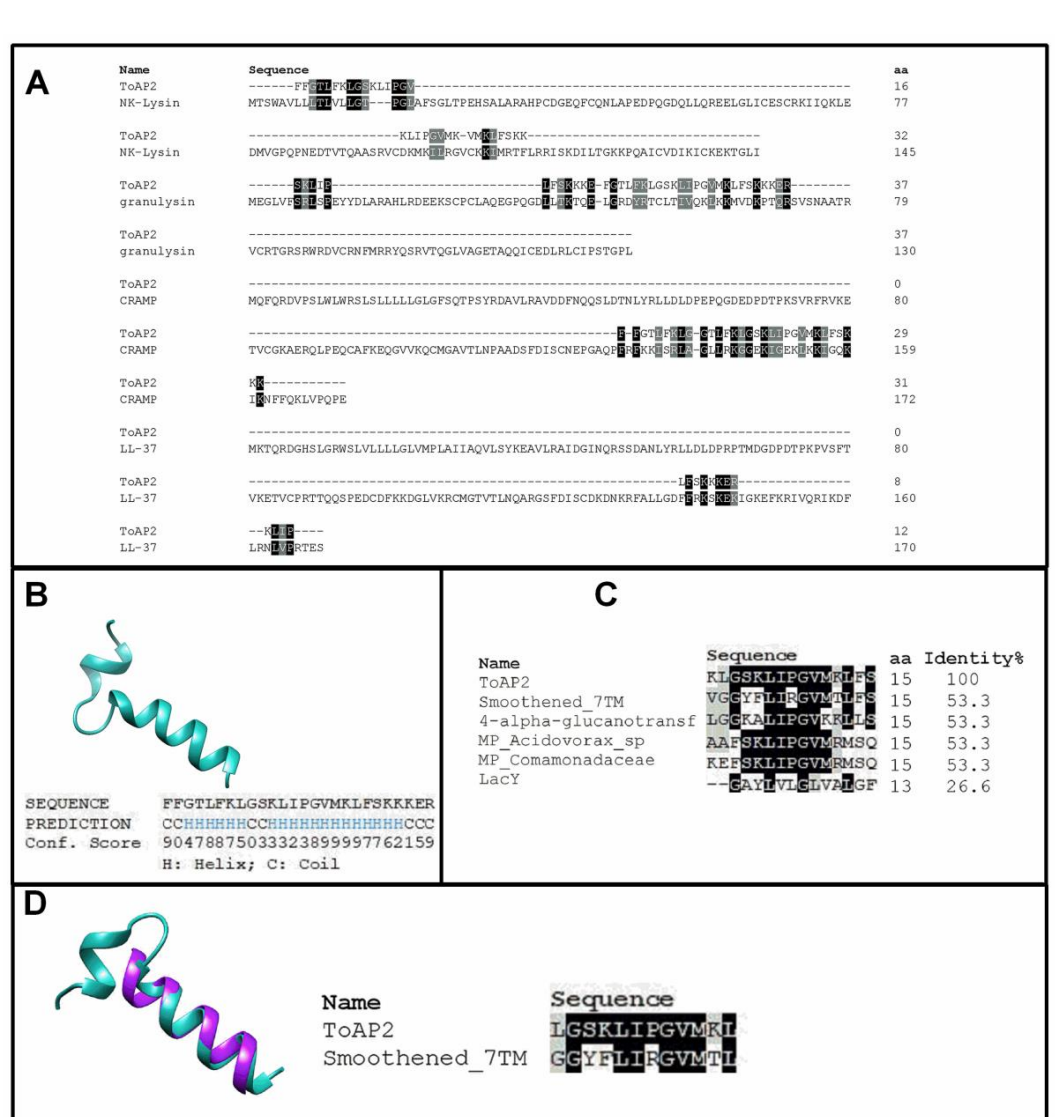
In silico analyses of ToAP2 and its conserved residues. (**A**) Pairwise alignment of ToAP2 and the proteins Granulysin, NK-Lysin, CRAMP, and LL-37, provided by the LALIGN online server. (**B**) Predicted secondary structure was provided by the PSIPRED tool, generated under sequence homology with 10 other sequences. (**C**) Multiple sequence alignment of ToAP2 with a conserved portion of membrane proteins, were provided by Clustal Omega. (**D**) Structural alignment with human smoothened 7TM protein, was generated by Chimera software and Emboss Needle.

ToAP2 is an AMP with proved efficacy against fungus, *Candida albicans* and *Cryptococcus neoformans* [19]. Its homology with antimycobacterial peptides, such as CRAMP (P51437.2), NK-Lysin (NP_001265684.1), Granulysin (NP_036615.2) and LL-37 (P49913.1) (Figure 1A), led us to study the activity of ToAP2 against an atypical mycobacterial strain, Mycmas. ToAP2, from the scorpion *Tityus obscurus*, has conserved residues with other registered antimicrobial peptides presenting an alpha-helix structure [20,21]. ToAP2 structures have been described previously as an alpha-helix peptide by circular dichroism. Since antimicrobial peptides mode of action are directly related with their secondary structure, for further structural homology analysis, its amino acid sequence was submitted to a bioinformatic program, the PSIPRED (Version 4.01, London, UK, 2016) online tool [22], and two alpha-helices were predicted (Figure 1B). Since many of the homologue AMPs do not have a three-dimensional structure deposited in the data bank, the ToAP2 structure was established with a similar sequence to other proteins. Interestingly, ToAP2 has a conserved region like several membrane proteins (4-alpha-glucanotransferase (OLA85926.1), MP_Acidovorax_sp (WP_007856673.1), MP_Comamonadaceae (WP_027101384.1), and LacY (EHX87228.1)) (Figure 1C) that spans from residue 8 to residue 25. A pairwise alignment made in the Chimera software (Version 1.12, Bethesda, MD, USA, 2017) package and the Emboss Needle tool (Version 6.0.0[M1] Cambridgeshire, UK, 2017) between ToAP2 and human smoothened 7TM protein (5V57_A) (60% identity to ToAP2) elicits the alpha-helix structure and similarity (Figure 1D).

### 2.2. ToAP2 Antimicrobial Effect on Mycmas

**Figure 2:**
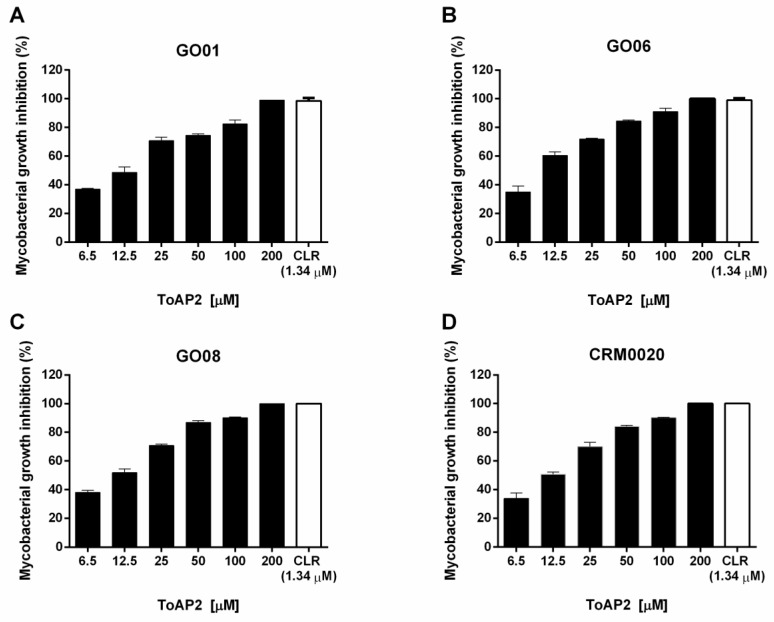
Growth inhibition of isolates and reference Mycmas strain by the scorpion peptide ToAP2. Peptide ToAP2 was used from 6.5 to 200 μM concentrations, against three clinical isolates (GO01 (**A**), GO06 (**B**), and GO08 (**C**)) and a reference strain (CRM0020 (**D**)). The percent inhibition was determined by comparison with control without peptide or any other antimicrobial. The MBC concentration was determined, for each strain, as the concentration that had no CFU after peptide treatment. CLR: clarithromycin.

The MBC of the peptide ToAP2 was determined by broth microdilution technique against Mycmas isolates (GO01, GO06, GO08) and a reference strain (CRM0020) (Figure 2). Among the concentrations tested, ToAP2 at 200 μM resulted in the greatest inhibition of growth. These results show that the ToAP2 peptide has the potential for the control of Mycmas growth, acting against all isolated and reference strains.

### 2.3. ToAP2 Antimicrobicidal Activity on Macrophages Infected with Mycmas

**Figure 3:**
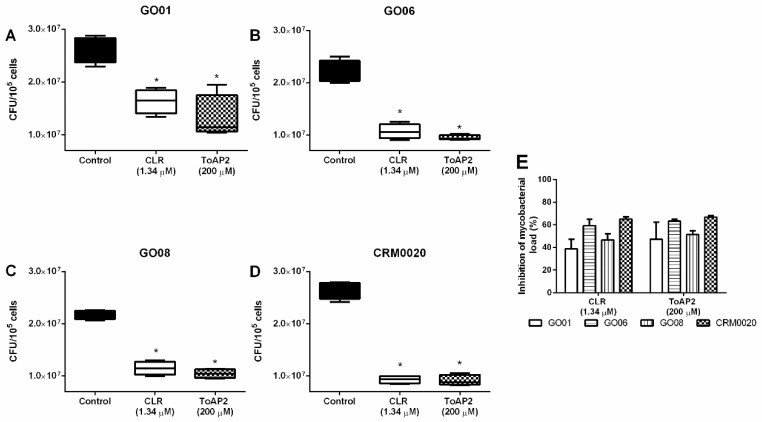
ToAP2 reduction of bacillary load in macrophages infected with Mycmas. Bone marrow-derived macrophages were infected with Mycmas strains (MOI 1:10) and then treated with ToAP2 MBC concentrations. Graph (**A**) represent GO01 strain, (**B**) GO06, (**C**) GO08, and (**D**) CRM0020. Graph (**E**) represents the percentage inhibition of bacterial load in all experiments and treatments. CFU: colony forming units. Differences between the means of the groups were determined by ANOVA and significant differences with *p* < 0.05 are shown with an asterisk (*), *n* = 4.

Mycobacteria mainly infect macrophages and the intracellular environment could prevent the action of antimicrobial molecules. Since the peptide ToAP2 showed antimycobacterial activity and has already been shown to have low toxicity [19], we have decided to test its efficacy in the treatment of infected macrophages. Bone marrow-derived macrophages infected with different clinical isolates GO01, GO06, and GO08 and the standard strain CRM0020 (Multiplicity of infection: MOI 1:10) and treated with ToAP2 at MBC (200 μΜ) reduced the bacillary load of macrophages infected with Mycmas (Figure 3). This reduction was similar to the inhibition induced by the control antibiotic (CLR, 1.34 μM).

### 2.4. ToAP2 Reduction of Bacillary Load in Spleen, Liver, and Lung of Mice Infected with Mycmas

**Figure 4:**
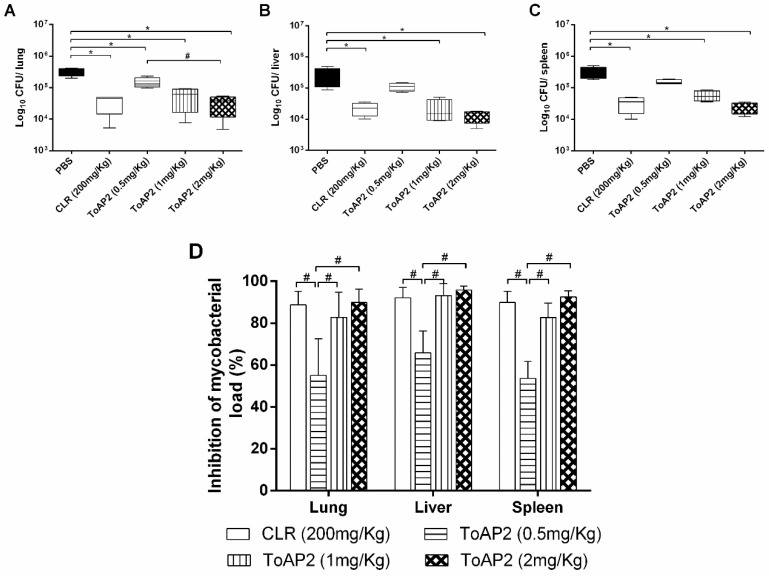
ToAP2 inhibits the bacillary load of Mycmas in organs of infected mice. IFN-γ KO mice were infected with Mycmas and treated with three different concentrations of ToAP2 (0.5 mg/kg, 1 mg/kg, and 2 mg/kg). After the treatment, the lung (**A**), liver (**B**), and spleen (**C**) were collected and the CFUs in these organs were evaluated. (**D**) shows the percentage inhibition of bacterial load in all organs and treatments. Differences between the means of the groups were determined by ANOVA and significant differences with the control, with *p* < 0.05 are shown with an asterisk (*). Significant differences between the other groups with *p* < 0.05 are shown with a hash symbol (#), *n* = 4.

IFN-γ KO mice were infected with Mycmas and, after 18 days of infection, were treated daily with ToAP2 at three concentrations (0.5 mg/kg, 1 mg/kg, and 2 mg/kg) for eight days. A significant reduction of the bacillary load in the lung (Figure 4A), liver (Figure 4B), and spleen (Figure 4C) was observed on mice treated with 1 mg/kg and 2 mg/kg doses, around 80%, the same reduction found when CLR was used as a treatment (Figure 4D). The highest ToAP2 dose used in vivo was 225 µM (300µg/mL). Guilhelmelli et al. (2016) evaluated the cytotoxicity and hemolysis of ToAP2. In our experiment, evaluating the hemolysis ranging from 6.25 to 1600 µM, only at higher concentrations (800 µM and 1600 µM) the peptide showed a hemolytic effect (26 and 28%, respectively). Using hemolysis data, the therapeutic index of ToAP2 for 10% hemolysis was 2.74.

### 2.5. In Vivo Chemotactic Activity of ToAP2 on Monocytes

Several AMPs were also characterized as chemotactic and, in some cases, immunomodulatory. Peptides LL-37 and CRAMP (mouse LL-37) presented chemotactic and modulating action over the immune response [23,24,25] and, therefore, potentiated their antimicrobial effect. To verify if the ToAP2A peptide presented chemotactic capacity, the peptide was inoculated into the peritoneum of C57BL/6 mice (2 mg/kg).

**Figure 5:**
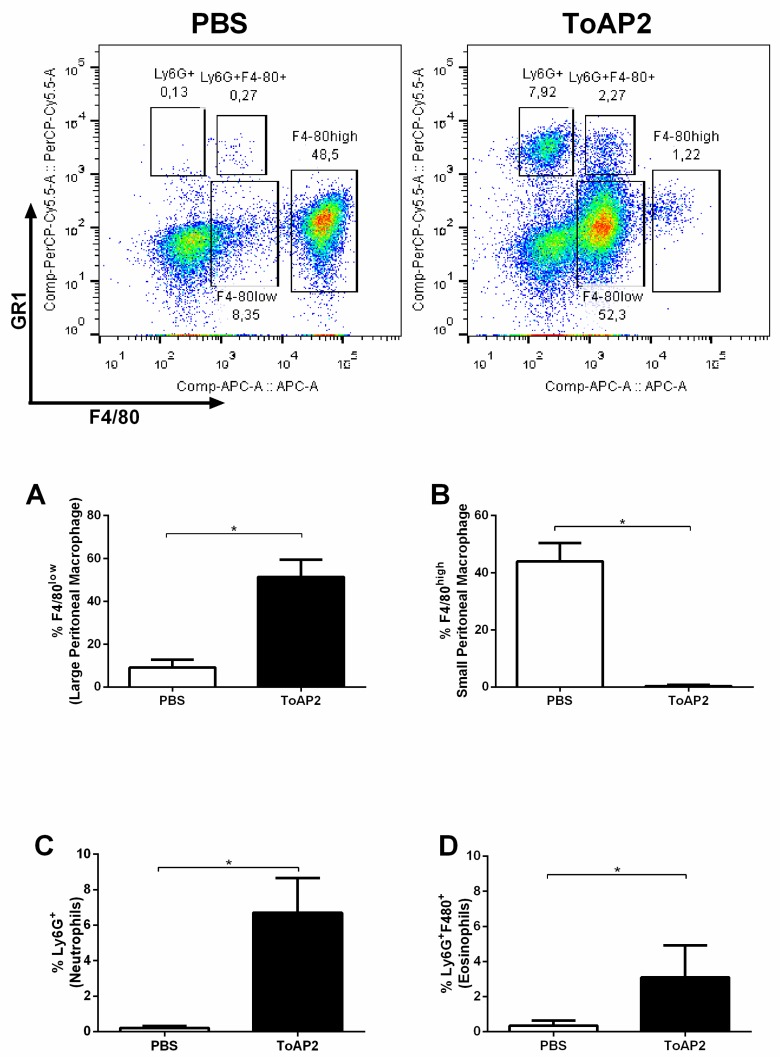
Chemotactic activity of ToAP2 in BALB/c mice peritoneum. Four animals from each group were injected intraperitoneally with 2 mg/kg of the peptide ToAP2 (in 100 μL) or with 100 μL of PBS. Twenty-four hours after the inoculation, mice were euthanized and the cells present in the peritoneum were evaluated. Differences between the means of the groups were determined by Student’s *t*-test and significant differences with the control, with *p* < 0.05, are shown with an asterisk (*), *n* = 4. Top figures show the dot plots of the acquired cells comparing GR-1 PerCP-Cy5.5 versus F4/80 APC. (**A**): Percentage of large peritoneal macrophages (F4/80^high^), (**B**): Percentage of small peritoneal macrophages (F4/80^low^), (**C**): Percentage of neutrophils (Ly6G+), (**D**): Percentage of eosinophils (Ly6G^+^F4/80^+^).

Peritoneal cells were evaluated 24 h after inoculation. Under normal conditions, peritoneal macrophages can be classified into large peritoneal macrophage (LPM) that are F4/ 80^high^ and small peritoneal macrophage (SPM) F4/80^low^ with a predominance of LPM. When the ToAP2 peptide was injected into the mice peritoneum, it induced a shift in cell populations, increasing the frequency of F4/80^low^ macrophages (Figure 5A) and reducing the percentage of F4/80^high^ macrophages (Figure 5B) in relation to the control group. ToAP2 was able to recruit two additional populations, inducing a greater migration of Ly6G^+^ cells (neutrophils) (Figure 5C) and, possibly, eosinophilic Ly6G^+^ F4/80^+^ cells (Figure 5D).

## 3. Discussion

Antimicrobial peptides have been shown to be promising antimicrobial molecules. For example, the NDBP-5.5 scorpion peptide derived from *Hadrurus gertschi* [26] and the Polydim-I wasp peptide from *Polybia dimorph* venom [11] have been described as capable of inhibiting growth, in vitro and in vivo, of Mycmas. In this work, we present a peptide, derived from the scorpion *T. obscurus*, ToAP2, which, in a previous study, demonstrated antifungal activity for different species of *Candida albicans* and *Cryptococcus neoformans* [19]. Here we demonstrate its antimicrobial activity in vitro, with better antimicrobial activity in vivo, equivalent to the antibiotic used to treat infections caused by this bacterium. The antimicrobial activity probably is due to its chemotactic activity for monocytes, neutrophils, and eosinophils.

In the in silico evaluation, it was possible to observe that ToAP2 presents conserved positively-charged or polar amino acid residues, being six lysine residues, one arginine, two serine, and one threonine. The presence of these amino acids correlates with the ability of peptides to interact with the negatively-charged cell membrane [21,27]. Additionally, the peptide also exhibited α-helix conformation, which has been correlated with the toroidal pore model of action and the ability to kill bacterial cells, by forming pores in the membrane and bacterial cell walls. However, the authors cannot confirm if such a mechanism was used by ToAP2 [28,29]. In alignment with other AMPs chosen for their antimicrobial activity against mycobacteria, ToAP2 showed a great similarity with the cathelicidin LL-37, which is also a chemotactic and immunomodulatory molecule [30]. Finally, it was also possible to verify the constitutional and conformational similarity with the 7TM protein, which is a transmembrane protein that is correlated with the G protein. The ability to interact with G protein-related receptors was also demonstrated for LL-37, CRAMP, and other natural peptides from frogs and wasps. Such peptides have been capable of interacting with the formyl-peptide (FRPL) receptor present in several cells [17,23,25]. In our study, however, it was not possible to confirm the interactions of ToAP2, and this should be addressed in future studies.

The ToAP2 peptide showed antimicrobial activity against Mycmas in vitro with 200 μM MBC, this MBC was also demonstrated in other scorpion-derived peptides, such as NDBP5.5 [26] and against other species of microorganisms, such as *Candida glabrata* [19]. Therefore, when used to treat intracellular infections, ToAP2 was as effective in treating infected macrophages as clarithromycin, in the same way as the NDBP5.5 peptide demonstrated by Trentini et al. (2017) [26].

When used to treat Mycmas-infected mice (IFN-γ KO) at doses of 1 and 2 mg/kg (Figure 4) it was also able to reduce bacterial load in the spleen, liver and lung, equivalent to the reduction generated by treatment with clarithromycin. These results are different from those presented for the NDBP5.5 peptide that, at 1 mg/kg dose, did not have the same capability to reduce the bacterial load in animal organs [26]. On the other hand, Rivas-Santiago et al. (2013) showed that LL-37 and CRAMP (peptide with higher identity with ToAP2, Figure 1A) were able to efficiently reduce the bacillary load of *Mycobacterium tuberculosis* at doses of 1 mg/kg, similar to that used in our study [31]. Thus, ToAP2 and CRAMP show related infection treatment capacities which, together with the alignment, suggest possible similarity in their mechanisms of action.

It was observed that ToAP2 induces the recruitment of F4/80^low^ macrophages and consequently reduces the F4/80^high^ macrophages (Figure 5). According to Ghosn et al. (2010) F4/80^high^ macrophages observed in the peritoneum are resident macrophages, whereas F4/80^low^ macrophages are derived from peripheral blood monocytes [32,33]. In a non-stimulated peritoneum, F4/80^high^ macrophages are the major population in relation to F4/80^low^, whereas when pro-inflammatory stimuli are used, such as LPS, zymosan, or thioglycollate, there is a change in the pattern of these cells and F4/80^low^ cells turn out to be the majority due to the influx of monocytes from the circulation while the tissue macrophages migrate to the omentum [32,33]. The stimulus with the ToAP2 produced on the cellular populations a similar effect to a pro-inflammatory stimulus because there was an increase of the F4/80^low^ cells, while there was a decrease of the F4/80^high^ cells. These data corroborate with the activity of other homologous peptides to ToAP2, such as LL-37 and CRAMP, which also have the ability to recruit monocytes and induce them to differentiate into macrophages with a pro-inflammatory profile. Contrary to this hypothesis, intra-peritoneal treatment with ToAP2 could have induced resident macrophage (F4/80^high^) population death, however, treatment of BMM (Figure 3) with ToAP2 did not show cytotoxicity against these cells. The peptide ToAP2 was also able to recruit neutrophils, characterized by GR1^+^F4/80^−^, and probably eosinophils (GR1^+^F4/80^+^) [34], however, peritoneal eosinophil present several other markers [32] that were not studied here, and this population should be further characterized. This chemoattractant activity was also demonstrated by CRAMP/LL-37 [25,35,36,37,38].

Together with the capability to recruit different cell populations, treating infection with AMPs should have the capacity to modulate the immune response. For example, CRAMP/LL-37 was demonstrated as having the capacity to induce leukotriene production by eosinophils [23,24]; and they were able to improve neutrophil survival (inhibiting apoptosis) [35], inducing chemokine and cytokines production, as well as improving NOS and ROS production [36]. Further, CRAMP/LL-37 were able to induce monocyte differentiation in pro-inflammatory cells and modulate the response to pro-inflammatory stimuli (such as LPS) [37,38]. This direct antimicrobial activity and the modulation of immune response can improve the capacity to kill the bacteria, as well as tissue repair and would healing, leading to resolve the infection, and was demonstrated to diminish mycobacteria infection [39]. However, our experiments were limited to the cell recruitment stage and the immunomodulatory activity of this peptide still needs to be evaluated. Thus, we conclude that ToAP2 have in vitro anti-mycobacterial activity that is improved in vivo due to chemotactic activity.

## 4. Material and Methods

### 4.1. Bioinformatics Analysis

The homology between the peptide ToAP2 and other antimycobacterial peptides was done through the pairwise alignment server, LALIGN. Prediction of the secondary structure was done by iTasser [22]. Multiple sequence alignment used the Clustal Omega tool (Cambridgeshire, UK) (https://www.ebi.ac.uk/Tools/msa/clustalo/). Three-dimensional prediction was obtained by the UCSF Chimera tool [40]. Emboss Needle was used for matching between ToAP2 and 7TM receiver (Cambridgeshire, UK) (https://www.ebi.ac.uk/Tools/psa/emboss_needle/).

### 4.2. ToAP2 Synthesis and Purity

ToAP2 was synthesized by Aminotech Development and Technology, through solid phase chromatography using an *N*-9-fluorophenylmethoxy-carbonyl (F-MOC) strategy and purified through RP-HPLC. Samples with high purity, containing more than 95% of the peptide, were used for biological experiments. Peptides were stored at −20 °C and dissolved in Milli-Q water plus 3% DMSO before each experiment. The sequence and purity was determined and described by Guilhelmelli et al. (2016) [19]. Briefly, the purity and sequence of ToAP2 were submitted to matrix-assisted laser desorption/ionization time of flight mass spectrometry (MALDI-TOF/TOF MS; UltraFlex III, Bruker, Daltonics, Germany), under reflector (MS) and LIFT^TM^ (MS/MS) positive modes. The monoisotopic molecular mass of the ion corresponding ToAP2 was determined by the ratio between *m*/*z* peaks in the spread profile (*m*/*z* ratio from 600 to 3000). Additionally, the interpretation of the MS/MS mass spectra and de novo sequencing ToAP2 was performed using FlexAnalysis 3.0 software (Bruker, Daltonics, Germany, 2013).

### 4.3. Bacterial Strain Preparation

For in vitro experiments three isolates of Mycmas GO01, GO06, and GO08 were selected from the collection bank of isolates in Brazil [2] and the reference strain CRM0020 [26]. These bacteria were grown in Mueller Hinton medium (MH, HIMEDIA) for three days at 35 °C. Soon after, the cultures were adjusted to 1.5 × 10^8^ CFU/mL (0.5 McFarland scale).

As it was not found great difference between the strains, for the in vivo experiment (intravenous infection), the Mycmas GO06 isolate was used. The suspension was diluted in PBS, 0.05% Tween 80, and adjusted to 10^6^ CFU/mL.

### 4.4. Animals

BALB/c mice (for bone marrow production) and IFN-γ KO (for the in vivo experiment), aged 6–8 weeks were obtained from the laboratory of the Institute of Tropical Pathology and Public Health (IPTSP/UFG). All animals were kept according to the recommendations of the National Council for Animal Experimentation Control (CONCEA), with food and water ad libitum and controlled temperature, humidity, and light cycle. The protocols were approved by the Committee of Ethics of animal use of the Federal University of Goiás (protocol number: 016/14).

### 4.5. Minimal Bactericidal Concentration (MBC) Evaluation

Determination of MBC was performed as described by Trentini et al. (2017) [26]. The lyophilized ToAP2 peptide was diluted in 3% DMSO, and Mycmas cultures (all three isolates GO01, GO06, and GO08; and the reference strain CR0020) were adjusted to 100 CFU/well in 96-well plates containing different concentrations of the peptide ToAP2 (6.5 to 200 μM) diluted in PBS. Plates were incubated for 24 h at 35 °C. As a control, untreated mycobacteria and mycobacteria treated with CLR (1 μg/mL–1.34 µM) were used. The minimal bactericidal concentration was performed by counting the bacterial colonies.

### 4.6. Antimicrobial Activity of ToAP2 in Bone Marrow Derived Macrophage Infected with Mycobacterium massiliense

The femurs of BALB/c mice were collected after euthanasia of the animals and the cells were extracted from internal bone lavage with RPMI medium (SIGMA, St. Louis, MO, USA). Cells were cultured in the presence of 10 ng/mL GM-CSF. After three days of cultivation, 10 ng/mL GM-CSF was added to the culture. The differentiated macrophages after four days of culture were counted and plated at 10^5^ cells/well in 96-well plates. Cells were infected with Mycmas [MOI: 10:1] for three hours. After this period, cell cultures were washed with RPMI-1640 medium (HIMEDIA, West Chester, PA, USA), supplemented with 10% Fetal Bovine Serum, 1 mM sodium pyruvate, 2 mM l-glutamine, and 2 mM non-essential amino acids.

Treatment of the cultures with 200 μM of ToAP2 diluted in the medium described above was performed, and after 24 h the bacillary load was evaluated. To evaluate the bacillary load, the cells were lysed mechanically with 200 mL of ice-cold deionized water and the lysate was plated on MH medium.

### 4.7. In Vivo Antimicrobial Activity of ToAP2 against Mycobacterium massiliense

IFN-γ KO mice were infected with 10^6^ CFU/animal with Mycmas by the endovenous route. After 18 days of infection they were treated for eight consecutive days, by the intraperitoneal route, with the peptide ToAP2 in three different doses (2, 1, or 0.5 mg/kg) or with clarithromycin (CLR 200 mg/kg) [11,26].

One day after the end of treatment the animals were euthanized and spleen, liver, and lungs were collected to determine the bacillary load in these organs. For the CFU count, the organs were macerated, diluted in PBS 0.05% Tween 80, and plated on MH medium. The percent reduction in bacterial load was calculated by the difference between the infection controls and the treated groups.

### 4.8. In Vivo Chemotactic Activity of ToAP2

To evaluate the chemotactic activity of ToAP2, four mice were inoculated intraperitoneally with 2 mg/kg of the peptide. After 24 h, the animals were euthanized, and the peritoneum cells were collected. A group only inoculated with PBS was used as the control.

For intraperitoneal lavage, animals were euthanized by cervical dislocation and the peritoneum was exposed with the aid of surgical scissors and surgical tweezers. Five milliliters of ice-cold PBS was injected into the peritoneum for cell release, followed by dorsal massage. With a syringe, the peritoneal lavage was aspirated and this was stored in a 15 mL polystyrene tube in an ice bath for further processing.

For flow cytometry cell recruitment evaluation, cells were centrifuged and labeled with anti-Ly6G-PercP (clone:RB6-8C5) and anti-F4/80-APC (clone:BM8) for 30 min. A total of 50,000 events were acquired in the BD FACS Verse (Universidade Federal de Goiás, Faculdade de Veterinária e Zootecnia) and analyzed in Flowjo Vx software (Version 10.0.7.2, FlowJO, Ashland, OR, USA, 2016).

### 4.9. Statistical Analysis

The results were tabulated using Excel and Prism software packages (version 7.0, GraphPad, La Jolla, CA, USA, 2017). The differences between the groups were assessed with a nonparametric Kruskal-Wallis test followed by Dunn’s post hoc test. Significant differences were considered when *p* < 0.05. All three repetitions of the experiments showed similar responses.
